# Below 3 Å structure of apoferritin using a multipurpose TEM with a side entry cryoholder

**DOI:** 10.1038/s41598-021-87183-1

**Published:** 2021-04-16

**Authors:** Yoko Kayama, Raymond N. Burton-Smith, Chihong Song, Naoya Terahara, Takayuki Kato, Kazuyoshi Murata

**Affiliations:** 1 National Institute for Physiological Sciences, Okazaki, Aichi 444-8585 Japan; 2 Terabase Inc, Okazaki, Aichi 444-0864 Japan; 3 Faculty of Science and Engineering, Chuo University, Bunkyoku, Tokyo, 112-8551 Japan; 4 Institute for Protein Research, Osaka University, Suita, Osaka 565-0871 Japan

**Keywords:** Biophysical methods, Microscopy, Structure determination

## Abstract

Recently, the structural analysis of protein complexes by cryo-electron microscopy (cryo-EM) single particle analysis (SPA) has had great impact as a biophysical method. Many results of cryo-EM SPA are based on data acquired on state-of-the-art cryo-electron microscopes customized for SPA. These are currently only available in limited locations around the world, where securing machine time is highly competitive. One potential solution for this time-competitive situation is to reuse existing multi-purpose equipment, although this comes with performance limitations. Here, a multi-purpose TEM with a side entry cryo-holder was used to evaluate the potential of high-resolution SPA, resulting in a 3 Å resolution map of apoferritin with local resolution extending to 2.6 Å. This map clearly showed two positions of an aromatic side chain. Further, examination of optimal imaging conditions depending on two different multi-purpose electron microscope and camera combinations was carried out, demonstrating that higher magnifications are not always necessary or desirable. Since automation is effectively a requirement for large-scale data collection, and augmenting the multi-purpose equipment is possible, we expanded testing by acquiring data with SerialEM using a β-galactosidase test sample. This study demonstrates the possibilities of more widely available and established electron microscopes, and their applications for cryo-EM SPA.

## Introduction

Cryo-electron microscopy (cryo-EM) single particle analysis (SPA) is a technique for reconstructing the three-dimensional structure of a biomacromolecule using projected images acquired with an electron microscope^1^ and was the subject of the Nobel Prize for Chemistry in 2017^2^. The technique has achieved tremendous progress by integrating various technologies^3^.

Advances in SPA have been mainly driven by improvements of electron microscope performance^4,5^, developments of electron beam direct detectors^6,7^, methods for three-dimensional structure reconstructions^8–11^, and automated acquisition via Leginon^12^, SerialEM^13^, or manufacturer software. In recent years, near-atomic resolution has been achieved^14–16^ which permits construction of atomic models without foreknowledge of the protein sequence. True atomic resolution at 1.2 Å has also been demonstrated more recently^17,18^.

All the above-mentioned techniques are indispensable for improving achieved resolution. However, focusing on the performance of the electron microscope, including electron source, the sample stage, and the detector is arguably the primary limiting factor. For example, autoloader stages such as those used in Titan Krios (Thermo Fisher Scientific) and CRYOARM (JEOL) microscopes demonstrate that multiple sample grids can be stored stably for a long period of time, the sample grid can be automatically transported, and data can be automatically collected without manual intervention. Such a sample stage is difficult to introduce later into a multi-purpose electron microscope and is currently only available pre-installed. Such electron microscopes are expensive and are currently only available at limited locations. As a result, competition for machine time is high. One solution is to reuse established equipment.

Optimisations of the microscope for SPA are often incompatible or non-ideal for other techniques for which the microscope could be used, such as electron tomography^19^, energy-dispersive x-ray spectroscopy (EDS)^20^, electron energy loss spectroscopy (EELS)^21^, scanning transmission electron microscopy (STEM)^22^ and microcrystal electron diffraction (microED)^23^. It is difficult to maintain many microscopes all individually optimised for different tasks in each institute. Therefore, it is desirable to be aware of how realistic mid- to high-resolution SPA is on multi-purpose TEMs.

While datasets exceeding 1,000 micrographs are now regularly collected in cryo-EM SPA^24^, this is unrealistic for manual data collection. Therefore, firstly, limited datasets (< 200 micrograph movies each) were manually collected with a highly symmetrized test specimen of apoferritin on two microscope-and-detector combinations which serve as multi-purpose (S)TEMs, both using Gatan 626-type cryo-specimen holders. Apoferritin^25,26^ is a relatively recent addition to the “benchmark” test samples for cryo-EM, since its compact size, spherical shape and octahedral symmetry previously presented difficulties in reconstructing at lower resolutions^27^. With advances in cryo-EM, its structural stability and high symmetry make it good candidate for quick testing of microscope hardware. Using a beta release of RELION 3.1 and < 300 micrograph movies, a 3 Å (global) resolution map of apoferritin was achieved in this study. We further examined optimized data collection conditions for each general purpose cryo-EM setting. The limited number of acquired micrographs is still useful to evaluate the ability to perform SPA analysis for high symmetry sample like apoferritin on particle count and reconstructions.

While not all multi-purpose TEMs can be equipped with automation software, the decrease in workload for the microscope operator presented by automation, combined with the ability to collect data more quickly, makes software-controlled data acquisition highly desirable. To demonstrate the utility of automation on data collection with a multi-purpose TEM which still requires manual cryogen maintenance, we secondly acquired a dataset of β-galactosidase using SerialEM, post-installed automated software^13^ and processed independently. Since β-galactosidase has a lower symmetry than apoferritin, D2 symmetry rather than octahedral, the particle count becomes more critical to achieve resolution. As a result, a 3.6 Å resolution map was reconstructed from approximately three-times more micrographs than manual operation acquired in one semi-automated session of data collection. This session took six hours with two replenishments of liquid nitrogen.

In this work we performed basic technical analysis of several factors important to cryo-EM data acquisition on two cryo-EM equipment setups. Then, we demonstrate the possibility of reusing existing equipment for cryo-EM through reconstructions of “benchmark” proteins across a range of imaging conditions. We discuss guidelines for the minimum setup for growth of the research field within general-access facilities.

## Results

### 3 Å resolution map reconstruction using a multi-purpose TEM with a side-entry cryo-holder

**Figure 1:**
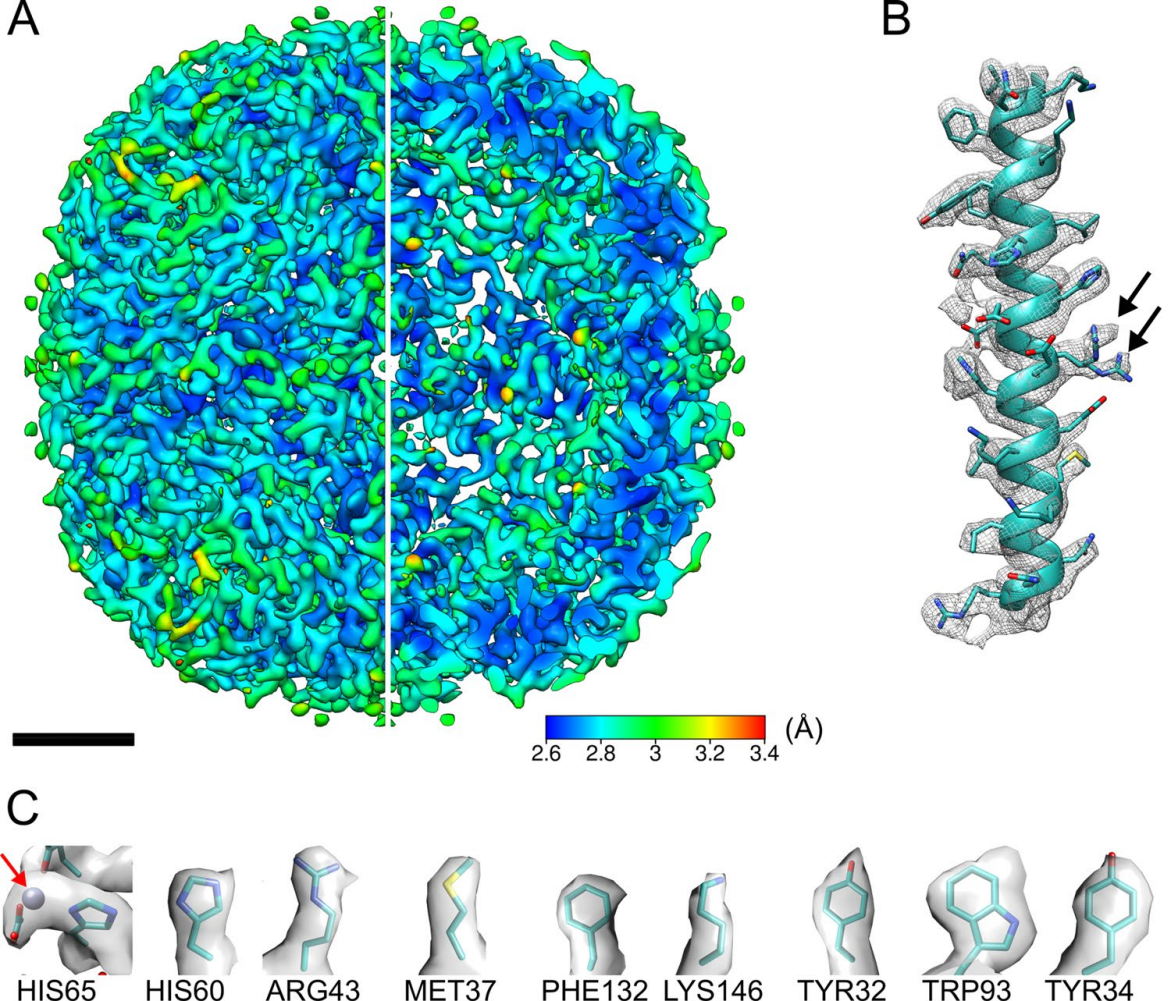
3 Å (global estimated resolution) cryo-EM map of mouse heavy-chain apoferritin. (**A**) Map coloured by local resolution; one quarter sliced away to allow visualisation of internal density. Scale bar 2 nm. (**B**) Representative helix (Leu48-Arg77) from one subunit, with PDB: 2CIH fitted. Map is contoured at 3σ. Black arrows show two rotamers of Arg63. (**C**) Nine representative sidechains. Red arrow shows a metal density.

**Figure 2:**
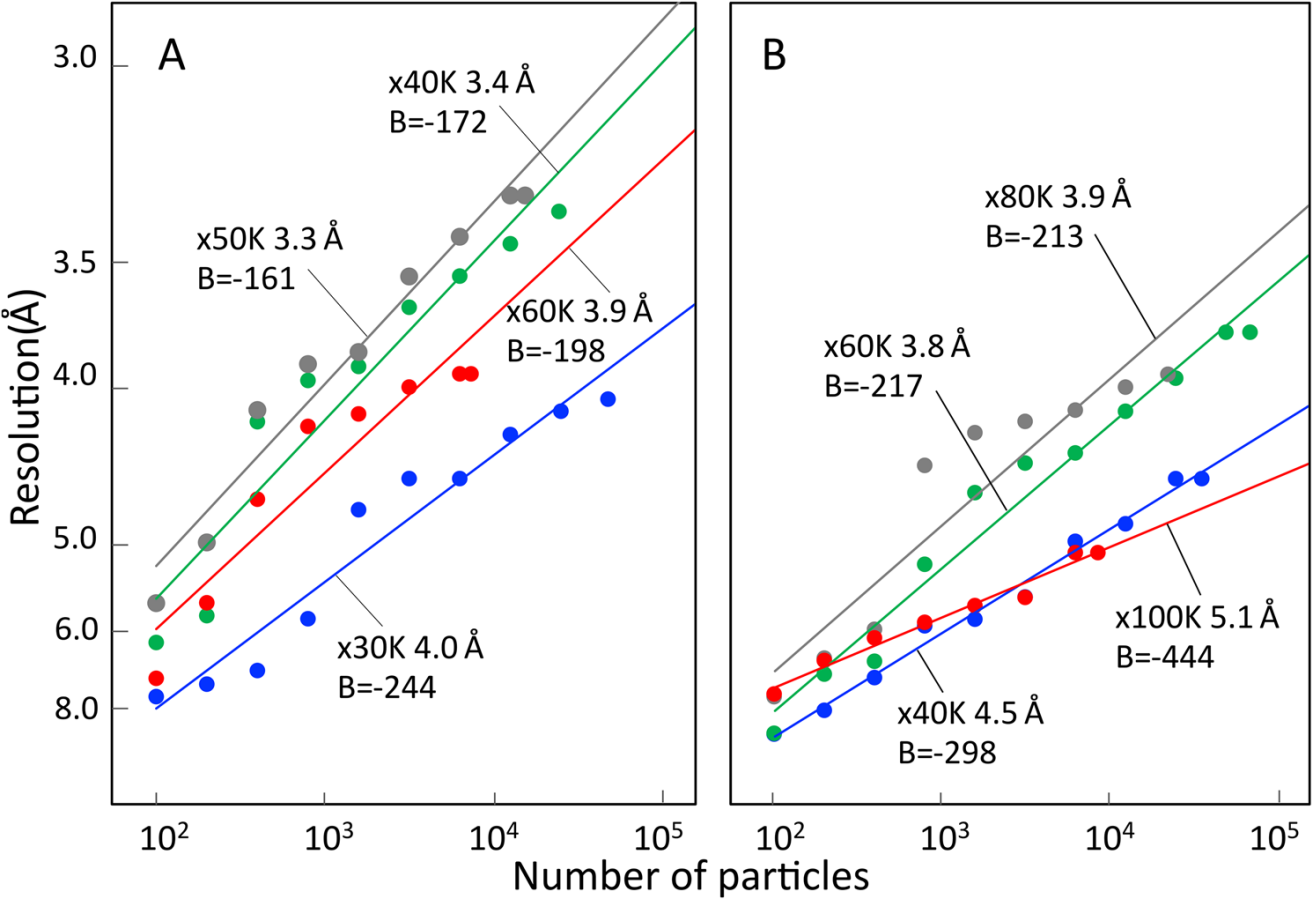
B-factor plots of all reconstructions. The points were calculated by *bfactor_plot.py* in RELION 3.0 at different magnifications. B-factor values are estimated from the fitted slopes. (**A**) Setting A and (**B**) Setting B. Magnifications are labelled appropriately.

With a limited dataset of 279 micrograph movies and using a beta version of RELION 3.1, we achieved 3 Å (global) resolution of apoferritin (Fig. 1) when estimated at the gold-standard (GS) (fully independent half-maps) Fourier shell correlation (FSC) (0.143) (Fig. S1A) using Setting A (a combination of JEM-2100F electron microscope and K2 Summit DED). This required a total of 57,057 particles to achieve. The calculated map-to-model (MM)-FSC is 3.3 Å (Fig. S1A), with an estimated B-factor of -156 (Fig. S1C), which is a minor improvement of that from the RELION 3.0 reconstruction (Table 1, Fig. 2). Local resolution estimated by the *blocres* module of Bsoft^28,29^ shows significant areas of the map between 2.6 and 2.8 Å (Fig. 1A). All helices were clearly defined (Fig. 1A,B) with some residues exhibiting two conformational states (Fig. 1B, marked with black arrows) although with one conformation dominant as the second was lost at higher map σ (Fig. S1B). In higher resolution regions side chains are clear (Fig. 1C) and densities could be assigned to metal atoms coordinated by side chains (Fig. 1C, marked with red arrow). It may be possible to assign water to some densities, but we erred on the side of caution with respect to interpreting potential water-related density.

**Table 1:** Details of each of eight acquisition conditions.

Data collection	EM Setting A				EM Setting B			
TEM magnification	30,000	40,000	50,000	60,000	40,000	60,000	80,000	100,000
Pixel scale (specimen)(Å/pixel)	1.25	0.93	0.75	0.62	1.43	0.95	0.71	0.62
Exposure time (s) (Adjusted by dose weighting during processing)	5	5	5	5	5	5	3	3
Dose per second (e^-^/Å^2^/s)	5.35	9.25	12.8	20.8	6.9	8.2	10.0	20.0
Exposure per frame (s)	0.2	0.2	0.2	0.2	0.2	0.2	0.2	0.2
**Single particle analysis**								
Particles per micrograph (avg.)	1,668	576	329	220	1,697	723	378	320
Acquired micrographs	100	101	153	112	100	150	100	183
Final micrographs	74	70	113	86	74	149	95	97
Initial particles picked	63,047	34,421	43,481	17,581	117,832	89.830	35,936	31,073
Final particle count	48,767	24,904	15,680	7,476	36,732	71,528	23,056	8,762
Resolution (Å) GS-FSC (0.143)	4.0	3.4	3.3	3.9	4.5	3.8	3.9	5.1
**Validation**								
Resolution (Å) MM-FSC (0.5)	4.5	3.6	3.8	4.5	5.3	4.3	4.2	6.1
Resolution “gap”	0.5	0.2	0.5	0.6	0.8	0.5	0.3	1.0
Rosenthal-Henderson (estimated B-factor)	-244	−172	−161	−198	−298	−217	−213	−444

### Imaging comparison of two EM settings

Photographs of one EM setup (Setting A; JEM-2100F, K2 Summit DED and Gatan 626 side entry cryo-specimen holder) are shown in Fig. S2. The second (Setting B; JEM-2200FS, DE-20 DED and Gatan 626 cryo-holder) has been shown previously^3^. Micrograph movies independently collected from the two EM settings were corrected for image drift and electron beam damage using the MotionCor2 algorithm^30^ as implemented in RELION 3^11^. Table 2 details the essential information for each equipment setting.

**Table 2:** Details of TEM Settings A and B.

	EM Setting A	EM Setting B
**Electron microscope**	JEOL JEM-2100F	JEOL JEM-2200FS
Gun type	200KV Schottky	200KV Schottky
Cs (mm)	2.0	4.2
Energy filter	None	Ω-type (15 eV zero-loss)
CLA (µm)	20	20
**Detector**	Gatan K2 Summit	Direct Electron DE-20
Pixel count	3708 × 3836	5120 × 3840
Pixel size (µm × µm)	5.0 × 5.0	6.4 × 6.4
Array size (mm)	18 × 19	33 × 25
Mag. factor	1.5	1.2
**Cryo-specimen holder**	Gatan 626 LN_2_	

**Figure 3:**
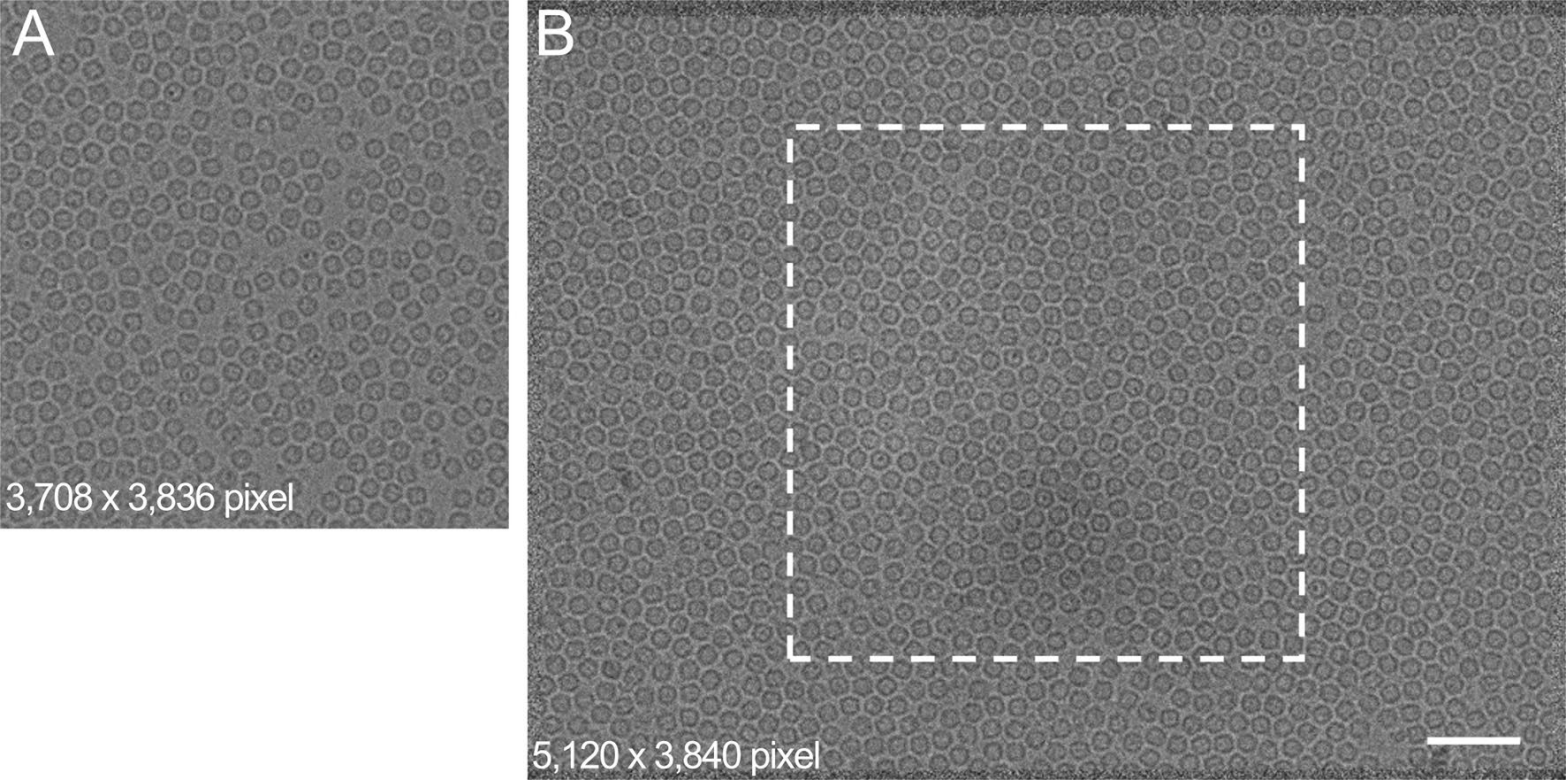
Comparison of micrographs from each microscope and detector combination. Representative micrographs from K2 Summit at 50,000 × (**A**) and from DE-20 at 50,000 × (**B**), each 2.4 µm under-focus. Micrographs scaled to equivalent size, scale bar 50 nm. White dashed box overlaid on image (**B**) highlights field of view difference between detectors. Micrograph dimensions are included in each figure. As a test sample, purified apoferritin was used. The number of particles included each micrograph were (**A**) 383, (**B**) 1,334, respectively. Absolute pixel dimensions are 5.0 × 5.0 µm^2^ for K2 Summit DED and 6.4 × 6.4 µm^2^ for DE-20 DED.

For comparison, representative cryo-EM images from each setup are shown in Fig. 3. In this figure, images were captured at 50,000 × magnification and displayed at the same particle scale. In both cases, the projected image of apoferritin particles was easily recognized with similar contrast. An area of the same absolute size as the image obtained with Setting A (K2 Summit detector) acquisition is shown on the Setting B (DE-20 detector) micrograph by a white dashed box (Fig. 3B), highlighting the difference in field of view. The total number of apoferritin particles in the example images were counted, totalling 383 particles in the Setting A micrograph and 1334 particles in the Setting B micrograph (Fig. 3).

We calculated the MTF and DQE curves (Fig. S3) in each case at 200 kV using a beam stopper and the FindDQE program^31^ to evaluate the total performance of two EM settings; the greatest difference between the curves was shown in the low frequencies. The calculated DQE value at lower special frequencies was > 80% for Setting A, but was reduced to ~ 35% when using Setting B. At frequencies above 3/4 Nyquist, the two detectors have similar response curves (Fig. S3). They show similar characteristics to the same DEDs on different microscopes^31–33^, which implies the detector is the major limiting factor for both EM settings.

When comparing Setting A and Setting B using Pt-Ir film^34^ (Fig. S4) the clarity of Thon rings in Setting A is immediately apparent in the power spectrum (Fig. S4A) and in the rotational average profile (Fig. S4B) to beyond the diffraction ring of 2.27 Å. Both Settings were observed at 100,000 × magnification, at which point Setting B was difficult to keep stable. This is manifest in the weaker oscillations in the CTF and diffraction ring at ~ 2.3 Å (Fig. S4C). Minor astigmatism of approximately 60 nm causes the blurring of the diffraction ring in Setting B upon rotational averaging, which while visible in the power spectrum is not as clear in the radial profile (Fig. S4D).

### Comparison of 3D reconstruction maps in different imaging conditions

**Figure 4:**
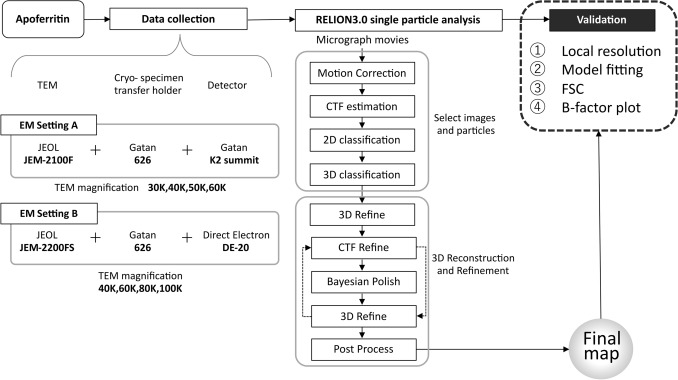
Processing workflow. General schematic of data acquisition and processing is described. Two electron microscope setups were used: JEM-2100F + Gatan 626 + Gatan K2 Summit detector (Setting A) and JEM-2200FS + Gatan 626 + Direct Electron DE-20 detector (Setting B). Each data set was processed using RELION 3.0 and evaluated with procedures shown in the figure. Further specifics of the equipment can be found in Table 2.

Eight cryo-EM maps of apoferritin with resolutions between 3.3 Å and 5.1 Å were obtained by standard operation (Fig. 4) of RELION 3.0 using different data sets as detailed in Table 1. Magnifications were chosen to cover a range of pixel scale calibrations widely reported in SPA literature and EMDB depositions^35^ (frequently 1 ± 0.5 Å/pixel) with as much overlap between the calibrated pixel scales as possible between settings A and B. The very latest and highest resolution data deposited^16–18^ were used typically 0.5 Å/pixel as the pixel scales, for which corresponding magnifications on both Setting A and B are 80,000 × and 120,000x, respectively, but are too high to maintain stability and a low enough dose rate for the sample when using the electron counting mode illumination condition with the standard procedure (recorded by five frames per second in each micrographs). In all final post-processing steps, a very soft mask (15 Å low-pass filter, 5 pixel expansion and 10 pixel soft edge) was used, which slightly reduces the resolution estimated by GS-FSC^36^ than when a less soft mask was used. We would prefer to underestimate the GS-FSC resolution than overestimate. Local resolution estimation is unaffected by this softer mask.

Using limited datasets described in Table 1, the highest resolution map was obtained from a data set acquired with Setting A at a magnification of 50,000 ×. The map coloured by local resolution^28,29^ is shown in Fig. S5. The best local resolution of ~ 2.8 Å was shown in the α-helices located inside the core. On the other hand, the disordered N-terminal of each subunit showed the lowest resolution of ~ 3.6 Å.

**Figure 5:**
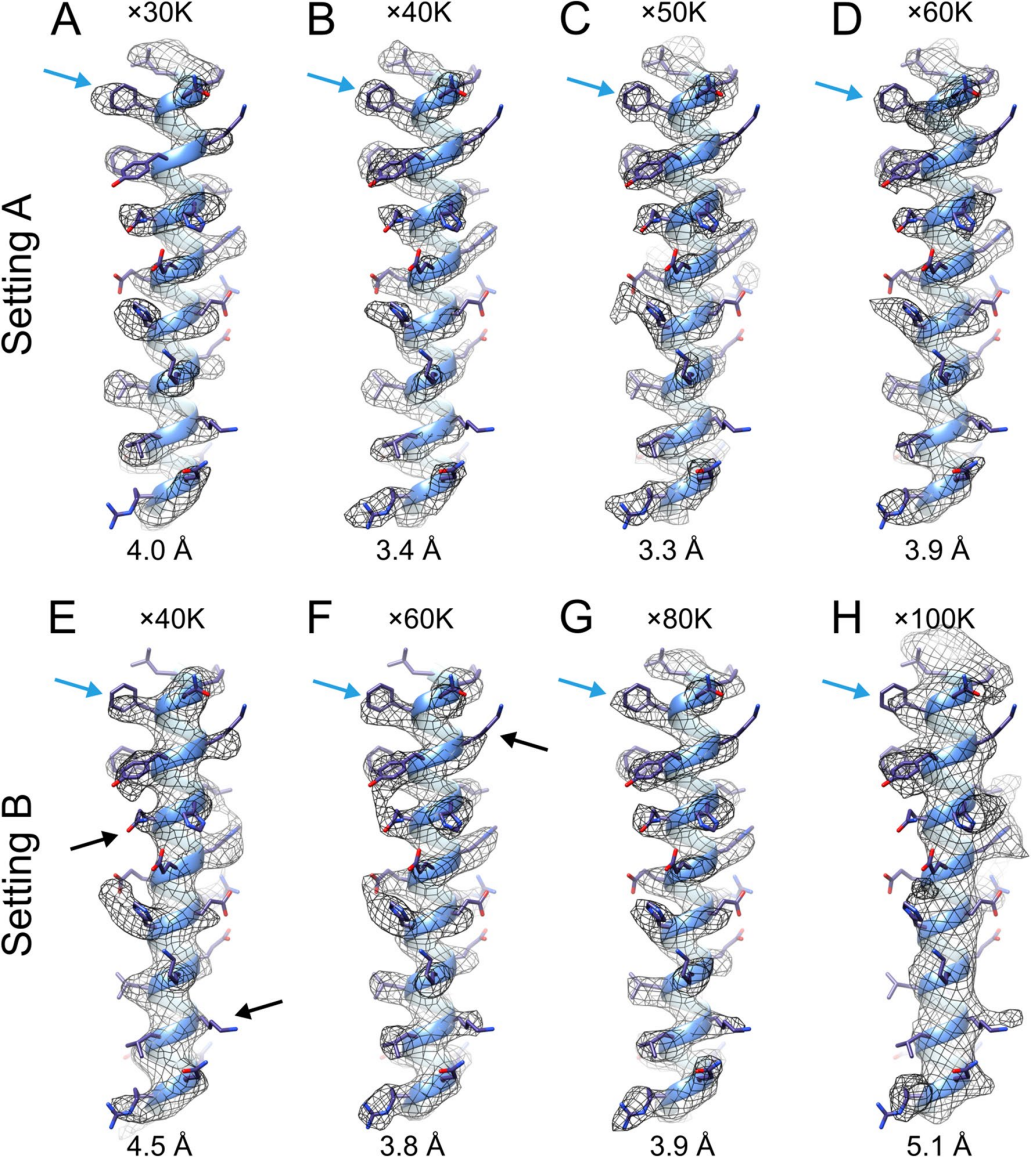
Density comparison between eight reconstruction segments. A representative α-helix (Leu48-Arg77) was extracted from each cryo-EM map and the corresponding atomic model (PDB ID: 2CIH) was rigid-body fitted to each map. The maps in the top panel are reconstructed from datasets acquired by Setting A, at magnifications of × 30 K (**A**), × 40 K (**B**), × 50 K (**C**), and × 60 K (**D**). The maps in each bottom panel are reconstructed from datasets acquired by Setting B, at magnifications of × 40 K (**E**), × 60 K (**F**), × 80 K (**G**), and × 100 K (**H**). The obtained resolutions are labelled in each map. Phe51 is indicated by blue arrows. Black arrows highlight exemplar sidechains which may present difficulties in identification.

A crystal structure model (PDBID: 2CIH)^26^ was fitted to the map and a single helix was extracted from each reconstruction to visualize map quality (Fig. 5). The highest resolution map (Fig. 5C) showed good agreement with the model to confirm the secondary structure and the side chains. Similarly, Fig. 5B also shows reasonable clarity for residue side chains. The slightly lower resolution maps (Fig. 5D,F,G,H) show moderately resolved side chains.

The best resolution map obtained using Setting B reported 3.8 Å at 60,000 × magnification, but there were some areas where the electron densities of the side chains cannot be clearly recognized; lysine and arginine residues provide examples (Fig. 5, marked with black arrows).

While it is still possible to determine larger side chains such as tryptophan, tyrosine and phenylalanine in maps of approximately 4 Å (Fig. 5A, marked with blue arrow), it is difficult to confirm the side chains in maps worse than 4.5 Å resolution (Fig. 5E). In maps with resolutions below 5 Å (Fig. 5H), it is not possible to reliably identify side chains and unstructured loops^37^.

### Comparison of data quality in different image conditions

**Figure 6:**
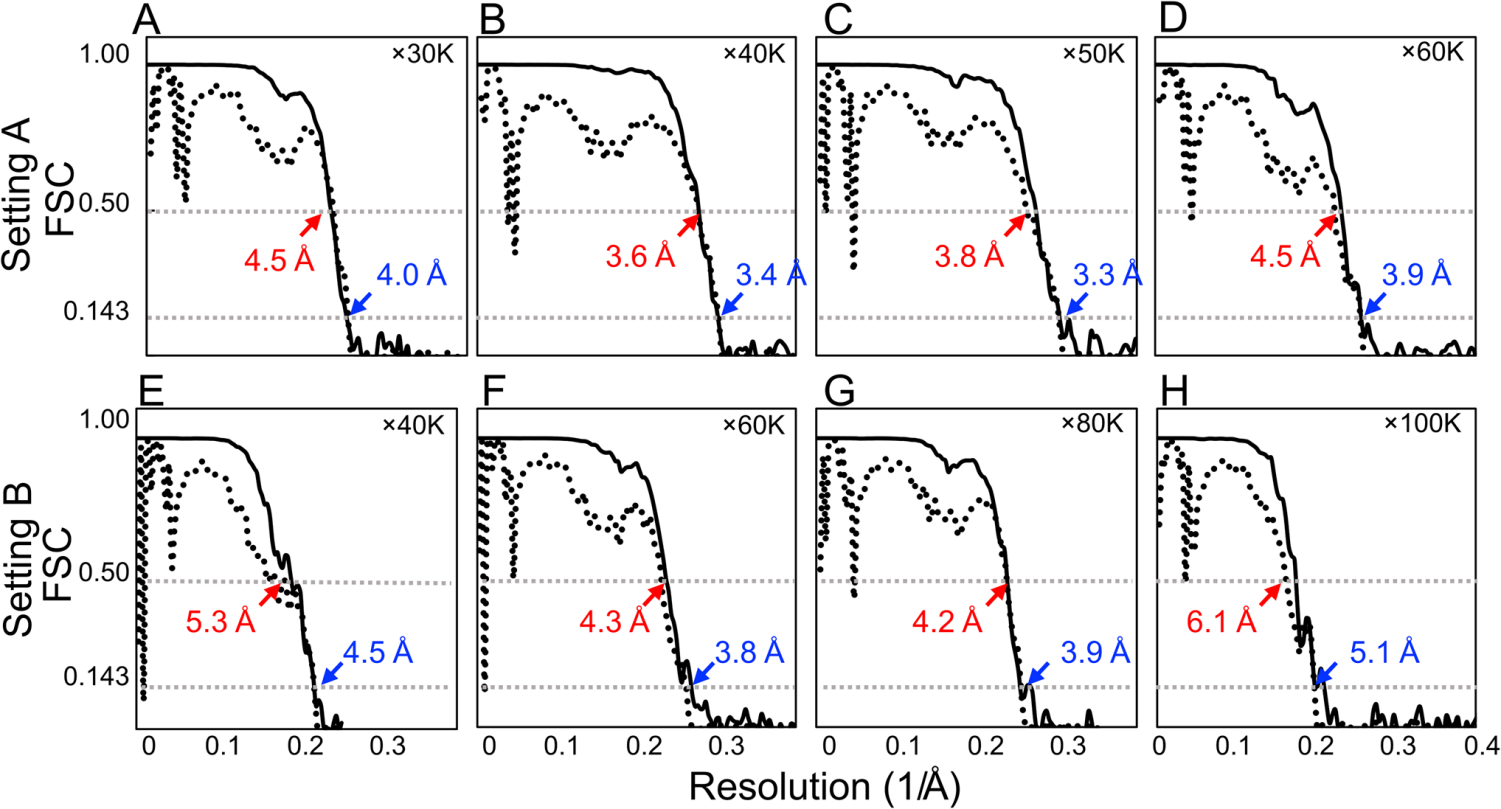
GS-FSC and MM-FSC plots of all reconstructions. GS-FSC (solid lines) was calculated from independent half maps, and the resolutions was estimated at 0.143 cut-off (blue labels). MM-FSC (dashed lines) were calculated between 3D reconstructions and maps calculated from the PDB model (2CIH), and the resolution was estimated at 0.5 cut-off (red labels). The experimental conditions are × 30 K (**A**), × 40 K (**B**), × 50 K (**C**), and × 60 K (**D**) magnifications using Setting A, and × 40 K (**E**), × 60 K (**F**), × 80 K (**G**), and × 100 K (**H**) magnifications using Setting B.

To verify the reconstructed maps, the GS-FSC and the map-to-model FSC (MM-FSC) were compared (Fig. 6). The gap between the two FSC values were listed in Table 1. The difference values (Resolution “gap” in Table 1) were averaged in each case, showing (average ± standard error) 0.45 ± 0.075 Å in Setting A and 0.65 ± 0.15 Å in Setting B. These smaller gaps between GS-FSC and MM-FSC of the Setting A correlate with the better-quality densities as shown in the maps (Fig. 5, Table 1).

**Figure 7:**
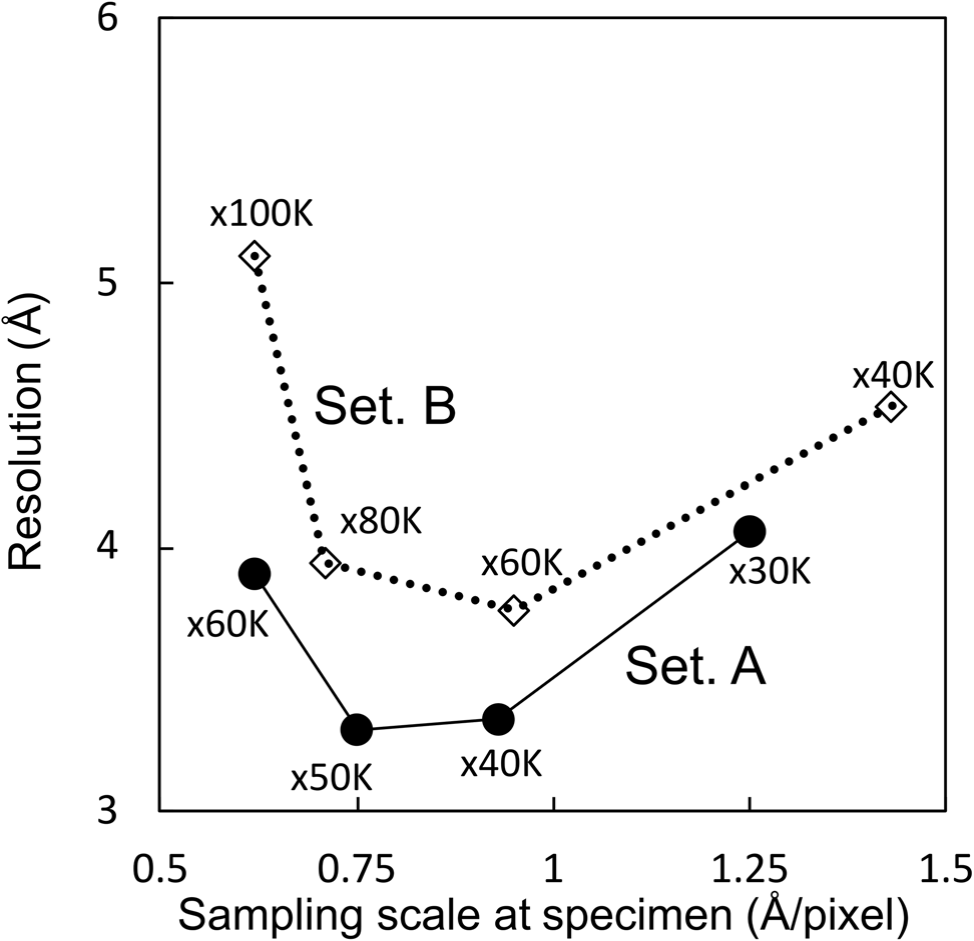
Plot of the achieved resolution of each magnification with different EM settings. The achieved resolutions of Setting A (solid dots) and Setting B (empty diamonds) at each magnification are connected by solid and dot lines, respectively. X-axis shows the sampling scale at the specimen.

The reported resolutions of Setting A between 30,000 × and 60,000 × magnifications showed a similar parabola shaped curve with Setting B between 40,000 × and 100,000 × magnifications (Fig. 7). The sampling scales at the specimen clearly overlap within these magnification ranges (Fig. 7, Table 1). The result suggests that there is an optimal magnification for high-resolution analysis in each EM Setting in our investigation. The highest resolution was obtained at 50,000 × magnification using Setting A, which corresponds to a sample scale of 0.75 Å/pixel (Fig. 7). Similarly, the highest resolution with Setting B was obtained at 60,000 × magnification, which corresponds to a sample scale of 0.95 Å/pixel (Fig. 7). The result suggests that the theoretically attainable maximum resolutions were 1.5 Å in Setting A and 1.9 Å in Setting B in the experimental conditions, respectively. However, many other factors will further restrict this Nyquist hard resolution limit. Intriguingly, changing the sample scales from the detector pixel size to that of the detector surface, these values both correspond to 0.15 Å/µm (Fig. S6) implying that the highest resolution is achieved when both JEOL 200 kV TEMs magnify the image at 0.15 Å/µm onto the detector surface. The difference of the highest resolutions’ nominal magnifications of 50,000 × on K2 summit and 60,000 × on DE-20 is caused by the difference of the detector mounting positions in each TEM. When calculating Rosenthal-Henderson (“B-factor”) plots (Fig. 2), Setting A has a clear advantage over Setting B. The 50,000 × Setting A dataset estimates the best B-factor (-161) with 40,000 × a close second (-172).

### Automated acquisition

**Figure 8:**
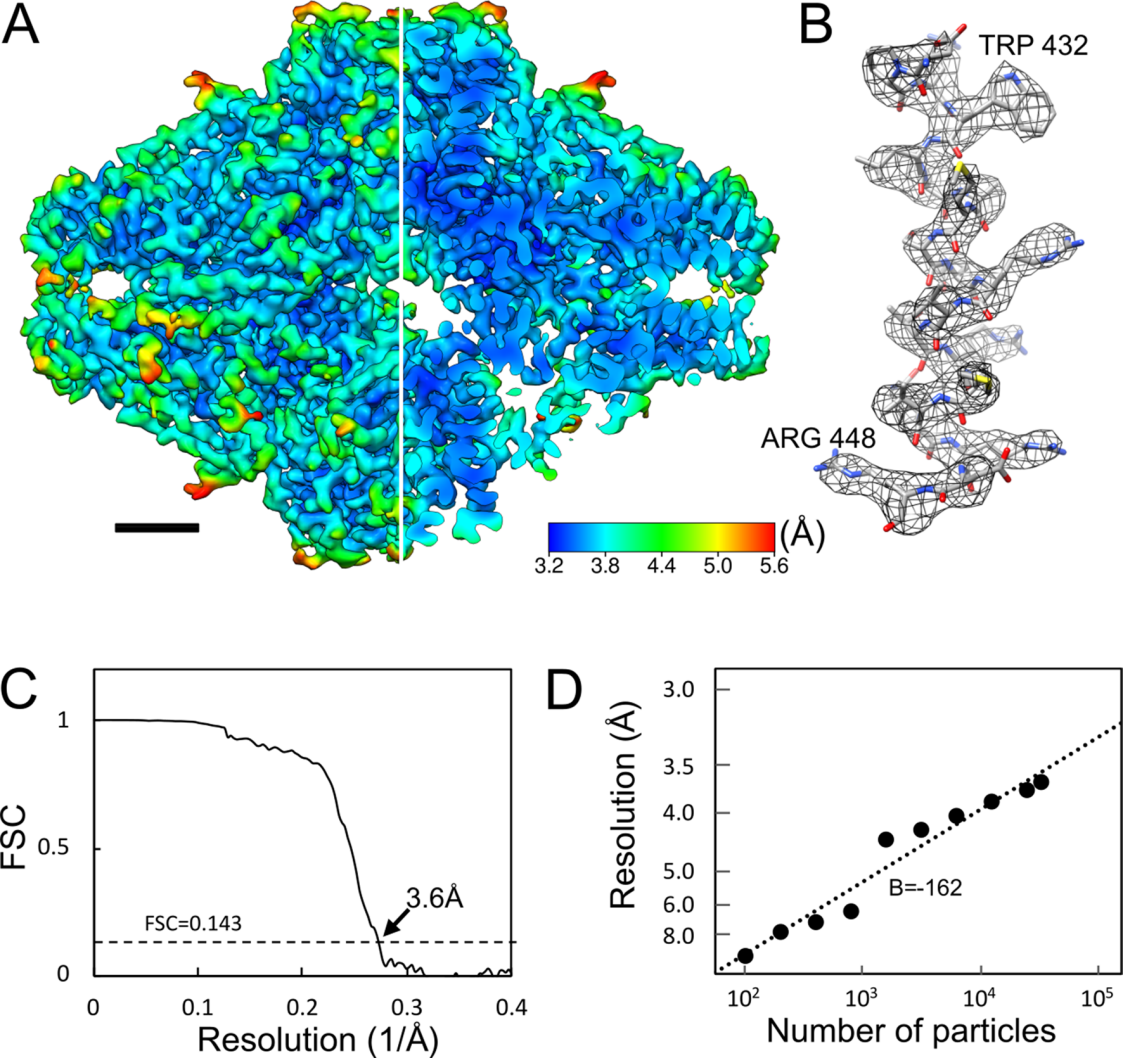
β-galactosidase reconstruction from Setting A using SerialEM automation software. Data was acquired in the same period (6 h) as other datasets, except via automation rather than manual operation. (**A**) 3.6 Å (global estimated resolution) reconstruction coloured by local resolution; one quarter sliced away to allow visualisation of internal density. Map is contoured at 5σ. (**B**) Representative helix (residues Asp429-Arg448) showing side-chain clarity. (**C**) GS-FSC curve. (**D**) Rosenthal-Henderson plot, estimating B-factor. Estimated B-factor is dependent on whether extremely low-particle-count reconstructions are included, which influences the overall B-factor estimate.

The purpose of this investigation is to demonstrate the ability of non-SPA-optimised microscopes. Adding automation via SerialEM does not de-optimise for other techniques as it can simply not be used during other acquisitions. Further, as stated in the introduction, automated acquisition is desirable due to increased speed of data collection and reduced load on operators. Thus, we examined the utility of automated acquisition on Setting A. β-galactosidase was used for the sample studied with automation in multi-purpose TEM to demonstrate that usable resolutions (Fig. 8A,B) may be achieved with lower symmetry samples compared to higher symmetry apoferritin, which requires more data to achieve similar resolutions.

Further details are listed in Table S1. In roughly the same amount of time—one half an operator day—that ~ 150 micrographs were collected manually, automation via SerialEM allowed acquisition of ~ 450. This is without any of the more advanced automatic collection methods such as beam shift acquisition^38^. Of these micrographs, 370 were deemed usable post-motion correction and CTF estimation. With automated collection, we achieved a 3.6 Å (Fig. 8C) resolution reconstruction of β-galactosidase (Fig. 8). Calculated B-factor (-162) is comparable to those of the apoferritin limited datasets of Setting A 40,000 × and 50,000 × (Fig. 8D).

## Discussion

After calculation of the Rosenthal-Henderson plots (Fig. 2) for all datasets, we estimated that collecting double or triple the number of micrographs of the original Setting A dataset at 50,000 × would achieve 3 Å global resolution. We collected a further 126 micrographs to complement the original 153 micrograph movies. This approximately doubled the number of usable particles but was slightly less than double the raw number of micrographs. Using RELION 3.0.8, this did not improve resolution achieved. Moving to a testing build of RELION 3.1 with improved calculation for optical parameters permitted a 3.0 Å reconstruction. Local resolution extended to 2.6 Å when calculated by *blocres*^28,29^. This resolution is sufficient to identify different sidechain rotamers (Fig. 1B, marked with black arrows). In addition, other sidechains also fit density well (Fig. 1C). After searching the Electron Microscopy Data Bank (EMDB)^35^ this is currently the highest resolution achieved when using a multi-purpose microscope and (non-autoloader) side-entry cryo-holder system. While now older, the Gatan 626 cryo-holder is in common usage around the world and can be considered a workhorse of many cryo-EM facilities. There are newer options, however, both from Gatan and from other manufacturers. Newer holders, such as the Gatan Elsa, or those developed to address long term unattended data collection, may yield better results. If expanding a current multi-purpose facility, all options should be examined.

The first evaluation metric of a cryo-EM reconstruction is the reported resolution and coincident FSC curve (Figs. S1A, 6). It is generally thought that the resolution improves as the magnification becomes higher. In our processing, this was untrue (Figs. 7, S6). Both Settings demonstrated higher resolution reconstructions as the magnification increases, until reaching the highest magnification at which point final resolution dropped. At lower magnification, Nyquist frequency imposes a hard limit on achievable resolution, thus improvement as magnification increases is easily explained. However, the abrupt loss of resolution at the highest magnifications must be caused by other factors. The most likely reasons are significantly decreased particle count, radiation damage or sample charging of the imaged area by the electron beam. Adjusting for the latter, however, is restricted by optimising beam conditions for the direct detector. Due to the requirements of the Gatan K2 Summit DED for electron dose in “counting” mode (~ 8 e^−^/(physical) pixel/s) the highest magnifications for each Setting should both be considered to have extremely high dose rates on the specimen. Grant and Grigorieff^39^ demonstrate that the first component of cryo-EM data to be eliminated by radiation damage is high resolution information. This would correlate with the lower resolution of the reconstructed map obtained at higher magnifications, although Setting B appears to suffer more impact than Setting A. However, maintaining stability of Setting B at 100,000 × was more difficult than Setting A at 60,000 × (Fig. 7) which may provide another explanation for the greater drop in resolution. As another reason, higher magnification means fewer particles visible per micrograph; it may be suggested that the manually collected, limited micrograph count (meaning fewer total particles) is the cause of lower resolution of the final map. However, B-factor plots can be used between reconstruction magnifications to show that this is not the case by measuring at 5000 particles for each magnification (Fig. 2). This occurs even though motion correction and dose weighting are performed. We hypothesise that adequate motion correction cannot be performed at a frame processing interval of 0.2 s (5 frames per second, fps) at higher dose rates due to the frame period containing motion blur beyond that correctable by B-factor sharpening. If all other factors playing a role in resolution were perfect, the attainable maximum resolutions are physically limited to 1.5 Å in Setting A (50,000 × magnification) and 1.9 Å in Setting B (60,000 × magnification) by the Nyquist frequencies in our experiments. While not a realistic purchase for a “multi-purpose” microscope, the high framerates of the Gatan K3^40^ and early results for the FEI Falcon 4^17^ show that higher framerate acquisition in counting mode will become more accessible and improve attainable resolution on multipurpose equipment in the future.

The best resolution was achieved at different magnifications in each EM setting (Figs. 5, 6, 7). Processing the “limited dataset” micrographs using RELION 3.0, the highest “limited set” resolution of 3.3 Å was obtained at 50,000 × magnification with Setting A, while the highest resolution of 3.8 Å was obtained at 60,000 × magnification with Setting B (Fig. 7, Table 1). When the sampling scales on each detector are compared respective to the detector surface area, the scale of 50,000 × on the K2 Summit detector (0.15 Å/µm) of Setting A corresponds to 60,000 × on the DE-20 detector (0.15 Å/µm) of Setting B. The difference is caused by the detector position in each microscope, in which the K2 Summit DED mounted under the camera room is lower than the DE-20 DED mounted in the film area. Interestingly, at this point the conditions for the best resolution were coincident between the two acquisition settings (Fig. S6). This would suggest that there is an optimal magnification for each microscope, regardless of detector sampling scale. This should be further investigated across a wider range of microscopes and detectors.

The map resolutions of Setting A are ~ 0.5 Å superior to Setting B, except for the highest magnifications (60,000 × (Setting A) and 100,000 × (Setting B)). Setting B provides the ability to collect superior numbers of particles from the same number of micrographs, which may prove advantageous in the case of larger or more heterogenous samples. However, the relatively lower Rosenthal-Henderson B-factors in Setting B (Fig. 2) suggests that the particle counts are not critical for resolution. We conclude that the superior performance of Setting A when compared to Setting B is primarily the result of the better detector performance of the K2 Summit DED^31–33^, which consequently improves the MTF and DQE of Setting A (Fig. S3) in addition to the Thon rings (Fig. S4), and secondly the result of the better spherical aberration (Cs: 2 mm) of the JEM-2100F microscope over the JEM-2200FS (Cs: 4.2 mm, which has a worse-than-normal Cs due to modifications necessary for the use of Zernike phase plate hardware).

When examining power spectra acquired using Pt-Ir film (Fig. S4), Setting A shows clear Thon rings distinguishable beyond 2 Å (Fig S4A, B). Setting B, on the other hand, demonstrates a considerably weaker power spectrum in which the last consecutive Thon ring is visible at 2.96 Å (Fig. S4C, D) and rotational averaging smears the Thon rings indicating minor astigmatism. The use of an Omega-type energy filter on Setting B contributes to usability of data (Fig. 3) by filtering out inelastically scattered electrons from the electron source. With two datasets taken without the Omega filter at 60,000 × and 80,000 × magnifications, micrographs were visibly lower quality and while they were acceptable for motion correction and CTF estimation, we struggled to proceed beyond 2D classification. Setting B is optimised for the use of the Omega filter, so removal of the filter may negatively impact the column optics more than expected. In our experiment, the influence of the Cs or detector performance would appear more significant to the results than the energy filter, although it may be more correct to say that the Omega filter compensates for the weaker DQE of the DE-20 direct detector. It would be interesting (and troublesome) to swap the detectors and/or objective lenses between microscopes and collect further datasets to identify whether objective polepiece (Cs), energy filtering or detector choice has the greater effect on final achievable resolution.

While the gold-standard FSC is a simple “one number” report of a cryo-EM reconstruction, it is, more precisely, simply a measure of correlation between two independently refined half-maps^36,41,42^. Map-to-model (MM) FSC is a comparison between a simulated volume generated by a fitted PDB model and either one of the two final half-maps of the GS-FSC or the post-processed (sharpened) full map. It is useful as a quality-of-fit metric for an atomic model, particularly if fitting the model via one half-map, and comparing against the second. The GS-FSC and MM-FSC for all Setting A maps are both superior to those of the Setting B maps (Fig. 6). This may come from the superior DQE curve (Fig. S3) and Thon rings of Setting A (Fig. S4) as we discussed in the previous section. However, there are some variations between two criteria in our reconstructions (Fig. 6). The gap between the GS-FSC and MM-FSC may just be an artefact of being at resolutions where there is some ambivalence in precise atomic positions (rotamers, etc.), sidechain identity (e.g.: between arginine and lysine), and ligands (e.g.: metals, small chemicals, and hydrated waters), as recent record-breaking reconstructions report no such GS/MM-FSC gap^17,18^. In both reports, atomic resolution is demonstrated and draws further attention to the fundamental differences between cryo-EM and x-ray crystallography regarding how the probe (quantum beam) interacts with the sample. Therefore, x-ray crystallography and cryo-EM model metrics may diverge again in the future.

We also evaluate the map quality by local resolution. This metric (recently combined with local filtering of a map) provides a finer-grain view of the quality of a reconstruction beyond that of the global or map-to-model FSC. With local resolution estimation, apoferritin shows highest resolution at the interaction face between the four bundled helices of each subunit; the contact points of the subunits and the external surfaces show lower resolution (Fig. 1A). The weakest point of an apoferritin reconstruction is the N-terminal of each subunit, like X-ray crystallography where unstructured terminus regions are difficult to resolve^26^.

All datasets show a roughly linear relationship (when plotted logarithmically) in B-factor plots; however, at a threshold of ~ 1000 particles some data points demonstrate a sudden drop before continuing in a linear fashion (Fig. 2). A recent report of a record-breaking resolution of apoferritin shows a linear relationship with as few as 54 particles^18^. From this we suspect that there may still be some heterogeneity in our final particle sets which only becomes evident at this threshold. Improving sample preparation technique such as gold grids, optimised ice thickness or better sample homogeneity would probably remedy this.

How valid is the use of a multi-purpose TEM for SPA? It will obviously not challenge the most expensive equipment combinations; however, this work demonstrates that even when imposing a limit on data quantity, it is possible to achieve usable resolutions for de novo structure determination. It is vital to understand the limits created by equipment: which magnifications are most stable, and the most easily optimised dose rate. The choice of detector will influence the rate at which data can be acquired and particles per micrograph. From the initial evaluation of Setting B, we did not expect to achieve sub-4 Å resolution, however, we were finally able to reconstruct the 3.8 Å resolution map of apoferritin by using recent image processing techniques. Therefore, the progress of software suites also supports the utility of multi-purpose TEM for SPA. With the improvements demonstrated here when reprocessing a dataset in RELION 3.1 versus RELION 3.0, we agree: demonstrating an improvement of ~ 10% through better image processing shows there are still improvements to come that are not hardware dependent.

When adding automated acquisition, quantity of data acquired during a similar period increases, although the percentage of good micrographs drops slightly compared to a skilled operator. The potential advantages for users regarding use of time and work environment cannot be overstated, although regular vigilance of cryogen levels is still critical as they are not automatically replenished. With manual acquisition, we normally acquire 30–50 micrographs per hour, although this is dependent on the detector used, total frames and save format. The operator included a settling time of ~ 5 s before collecting each micrograph. With automation, we collected ~ 75 micrographs/hour (Table S1) using the MRC format, with the same 5 s settling time. A more speed-optimised focussing strategy, faster detector, and/or beam shift acquisition, removing the necessity of a stage shift for each acquisition would increase this further. As collection rate increases, the impact of recording a greater percentage of unusable micrographs, whether by empty holes, protein aggregation or ice contamination decreases. The β-galactosidase dataset collected using SerialEM automation software (Fig. 8) demonstrates that acquisition of lower symmetry protein complexes is viable for achieving usable datasets in multi-purpose TEM.

We tested 200 kV multi-purpose TEMs for SPA in this study. However, there has been interest recently in lower accelerating voltages for SPA microscopes as image contrast improves as accelerating voltage decreases. Naydenova et al*.* demonstrated reconstructions on a 100 kV TEM with a custom DED^43^. Being able to carry out SPA on 120KV or similar TEMs would further open multi-purpose facilities. Unfortunately, current lower voltage microscopes are not ideal for SPA as commercial DEDs which can operate at 80-120 kV are not available. When commercially viable “low voltage” detectors become available^43^, this may again change the cryo-EM SPA landscape in the way that DEDs did originally^44^.

For SPA of varieties of samples using a multi-purpose TEM, the limiting factor is the ability to collect large volumes of quality data. Thus, we would recommend a direct detector as a reasonable minimum for cryo-EM SPA data analysis in “general purpose” EM facilities. Depending on the facility, a detector with a wider field of view may prove adventitious, although the potential increase in resolution of a detector with elevated DQE should not be discounted. Accompanying this with automated acquisition would improve the utility of SPA in such a facility greatly, but efficacy would depend on sample preparation, stability of TEM, and operator's skill. By achieving a global resolution of 3 Å with a multi-purpose TEM and limited datasets, we hope that we will encourage potential users of cryo-EM SPA who do not have access to state-of-the-art facilities.

## Materials and methods

### Cryo-electron microscopy

High-symmetry (24-fold/octahedral) mouse heavy-chain apoferritin was used as a test specimen, which was the generous gift of Dr. H. Yanagisawa, University of Tokyo^16,17,45^, and was supplied already purified. β-galactosidase (SIGMA-ALDRICH, St. Louis, MO) was purified by gel filtration on a Superdex-200 size-exclusion chromatography column connected to an ÄKTA FPLC apparatus (GE Healthcare Bio-Sciences, Piscataway, NJ) with an elution buffer comprised of 25 mM Tris (pH 8), 50 mM NaCl, 2 mM MgCl_2_ and 1 mM TCEP. An aliquot of either apoferritin or β-galactosidase sample solution was applied to standard molybdenum Quantifoil grids R1.2/1.3 (Quantifoil Micro Tools GmbH) and vitrified by rapid plunging in liquefied ethane using a Vitrobot Mark IV (Thermo Fisher Scientific) at 95% humidity and 4 °C. For apoferritin, the frozen grid was mounted on a Gatan 626 cryo-transfer specimen holder at liquid nitrogen temperature and loaded into either a JEM2100F microscope (JEOL) equipped with a K2 Summit direct electron detector (DED) (Gatan Inc) (Setting A) or a JEM2200FS microscope (JEOL) equipped with a DE-20 DED (Direct Electron LP) (Setting B). For β-galactosidase, the grid was mounted on a Gatan 626 cryo-transfer specimen holder and loaded into Setting A. Magnifications were varied and are detailed in Table 1. Both microscopes were operated with thermal Schottky electron source at 200 kV. In the case of JEM2200FS, an Omega-type energy filter was used with a slit width of 15 eV. Spherical aberrations of each pole piece were 2.0 mm (JEM2100F) and 4.2 mm (JEM2200FS). The illumination conditions were optimized for K2 Summit counting mode (8 e-/pixel/sec on detector) and maintained through DE-20 acquisition for comparison purposes via low dose acquisition, although at 80,000 × and 100,000 × magnification the dose rate for DE-20 acquired data was considered very high. Further details can be found in Table 1. Using SerialEM, a montage of the grid was created at 100 × magnification in "Low mag" mode and grid squares with lowest amorphous ice and uniform vitreous ice thickness were selected for acquisition. A second montage of the selected grid square was created at 250 × magnification in "Low mag" mode. From this, a grid of acquisition points was created, allowing automated recording of each hole. Micrograph movies were collected at 40,000 × magnification in "Mag" mode by moving the stage for each hole, auto-focussing every 5 holes. A template image of the hole at 3000 × magnification was used for adjusting the hole position accurately in "View" mode in SerialEM. Further details are described in Table S1. Movies were collected over a minimum of 3 s at 5 fps (frames per second) in both detectors.

### Image processing

Movies were motion-corrected using the MotionCor2 algorithm^30^ as implemented in RELION 3^11^ using dose-weighting and patch correction with a 5 × 5 grid for K2 Summit DED and a 5 × 3 grid for the DE-20 DED. The contrast transfer function (CTF) was estimated by CTFFIND4 (4.1.10)^45^. Particles were initially picked by either a) using the RELION 3 LoG-based (Laplacian of Gaussian) auto picker^11^ or b) manually picking 100–200 particles, as the shape of apoferritin means that more particles are not needed for autopicking. The box size was determined so that one edge was between 180 and 240 Å, and extracted particles were 2D-classified. Good classes were used as a reference for a second round of auto picking. The extracted particles were sorted against the autopicking references and the worst scoring particles discarded, after which they were subjected to 3D classification using a map generated ab initio using the cisTEM^8^ algorithm as a reference. The best class(es) were selected, and a map was refined via Refine3D followed by postprocessing. For further optimization, CTF refinement and Bayesian polishing were performed on the obtained input particles, and Refine3D was performed again in cycles to obtain the final map. Local resolution was calculated using *blocres* (with default settings but defining symmetry) from the Bsoft package^28,29^. The procedure is summarized in Fig. 4. Processing in RELION 3.1 proceeded in a similar fashion except for the particle sorting step, which has been removed from the GUI. Visualisation of 2D and 3D images were carried out using RELION^11^, Fiji^47^ or UCSF Chimera (1.11.2)^48^ depending on dimensionality.

### Model fitting and map validation

An x-ray crystallographic-derived atomic model (PDBID: 2CIH) was fitted to the eight maps obtained with different data acquisition conditions using the “fit in map” function in UCSF Chimera (1.11.2)^48^. The maps were segmented using SEGGER (v1.4.9)^49^ and an α-helix corresponding to residues 48–77 was visualized for each map. FSC curves of each of the data sets were calculated. The correlation between half-maps (GS-FSC)^34^ was estimated by 0.143 cut-off^50^, and the correlation between map and atomic model (PDB ID: 2CIH) (MM-FSC) was calculated with 0.5 cut-off. The PDB-based atomic model map was generated using UCSF Chimera's “molmap” function^48^ at the GS-FSC resolution of each reconstructed map. The CTFFIND estimated resolution was assessed by analysis of the logfiles generated by CTFFIND^46^. B-factor plots were calculated by running an appropriately modified copy of RELION's script, bfactor_plot.py. The script randomly selects subsets of particles from each data set and executes Refine3D and Postprocess steps. Plotting the natural logarithm of each particle subset against the inverse of the squared resolution for each refinement allows estimation of particle and dataset quality by linear fit correlation^11^.

### Assessment of TEM performance

DQE curves were measured using the shadow of a beam stopper. The obtained data was processed by FindDQE^31^. Thon rings are compared with two TEM settings by using FFT of Pt-Ir micrograms with the same data acquisition condition. Radial profiles were generated by Gatan Microscopy Suite 3 (Gatan, Inc.).

### Structural data

The cryo-EM reconstructions of all post-processed maps and half maps have been deposited in the EMDB under the accession codes: EMD-30096 (3 Å Setting A 50,000 × , RELION 3.1), EMD-30101 (Setting A, 30,000 × , RELION 3.0), EMD-30100 (Setting A, 40,000 × , RELION 3.0), EMD-30098 (Setting A, 50,000 × , RELION 3.0), EMD-30099 (Setting A, 60,000 × , RELION 3.0), EMD-30103 (Setting B, 40,000 × , RELION 3.0), EMD-30105 (Setting B, 60,000 × , RELION 3.0), EMD-30106 (Setting B, 80,000 × , RELION 3.0), EMD-30107 (Setting B, 100,000 × , RELION 3.0), EMD-30095 (Setting A, 40,000 × , RELION 3.0, Serial-EM acquisition, β-galactosidase).

## Supplementary Information

Supplementary Information.
