# Supplementary material for: Below 3 Å structure of apoferritin using a multipurpose TEM with a side entry cryoholder
Source: Sci Rep. 2021 Apr 16;11:8395. doi: 10.1038/s41598-021-87183-1 (PMC8052451; doi:10.1038/s41598-021-87183-1)
Supplement: Supplementary file 1 — Supplementary Information. [file 41598_2021_87183_MOESM1_ESM.pdf]

Supporting information

Below 3 Å structure of apoferritin using a multipurpose TEM with a side entry cryoholder

Yoko Kayama[1,2]*, Raymond N. Burton-Smith[1]*, Chihong Song[1], Naoya Terahara[3], Takayuki Kato[4] and Kazuyoshi Murata[1$]

[1] National Institute for Physiological Sciences, Okazaki, Aichi, 444-8585, Japan

[2] Terabase Inc., Okazaki, Aichi, 444-0864, Japan

[3] Faculty of Science and Engineering, Chuo University, Bunkyoku, Tokyo, 112-8551, Japan

[4] Institute for Protein Research, Osaka University, Suita, Osaka, 565-0871, Japan

*These authors contributed equally to this work.

[$]Correspondence email: kazum@nips.ac.jp

Table S1 Details of acquisition conditions for β-galactosidase via SerialEM.

Microscope	Setting A
TEM magnification	40,000×
Pixel scale (specimen)(Å/pixel)	0.93
Exposure time (s)	5
Dose per second (e^-/Å^2/s)	10.6
Exposure per frame	0.2
Micrographs per hour (SerialEM)	~75
Data collection time (h)	6
Total micrographs collected	451
Pause for refilling LN_2	2 (10 min. each)
**Single particle analysis**	
Particles per micrograph (avg.)	381
Number of micrographs used	370
Final particle count	50,523
Resolution (Å) GS-FSC (0.143)	3.6
**Validation**	
Resolution (Å) MM-FSC (0.5)	4.2
Rosenthal-Henderson (estimated B-factor)	-162

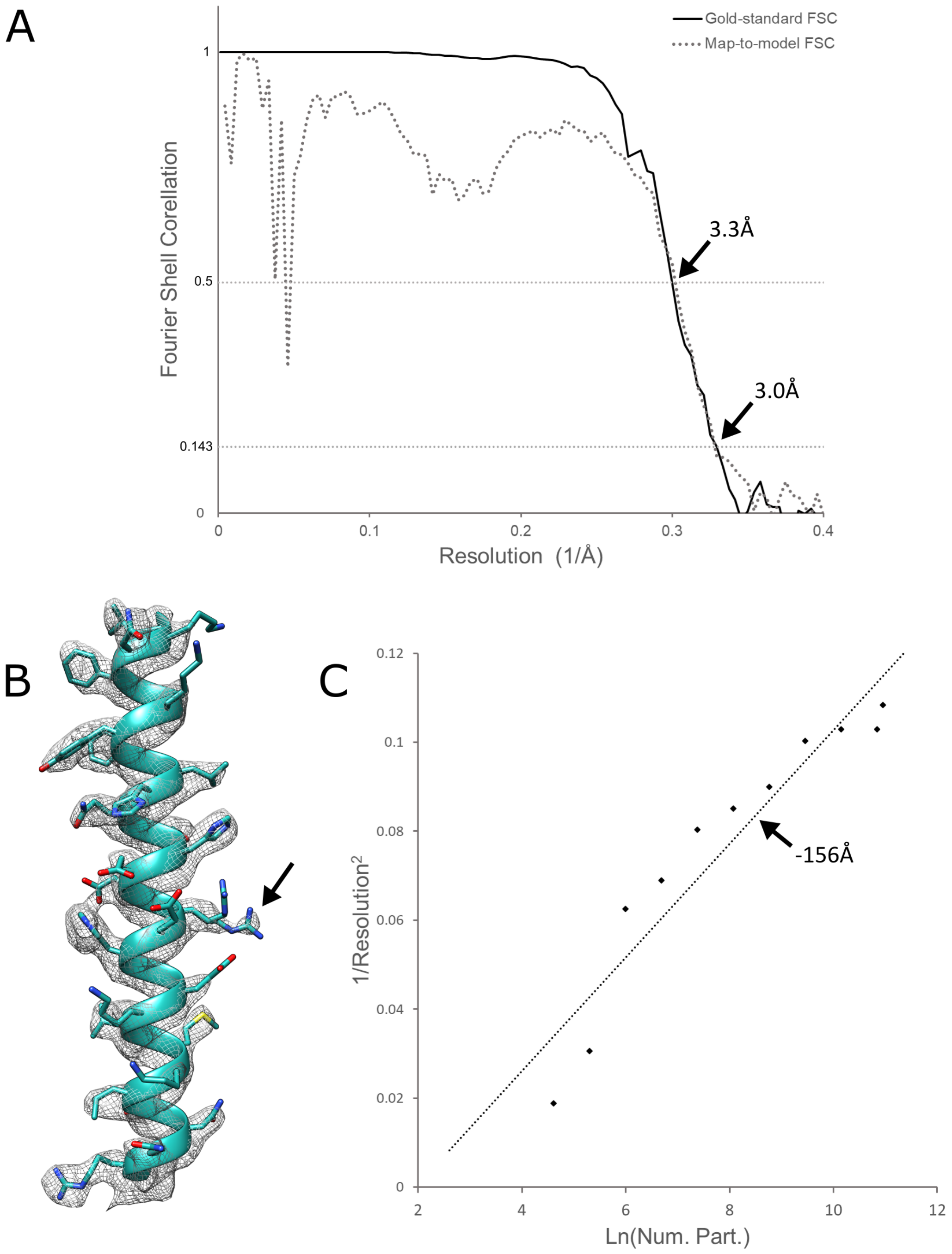

Fig. S1. Additional data for Fig. 1. A) Gold-standard FSC (black line) and Map-to-model FSC (dashed line) for 3 Å (global estimate) apoferritin reconstruction. B) The helix from Fig. 1B, contoured at 5σ rather than 3σ (Fig. 1B), permitting visualisation of the loss of one of the Arg63 rotamer densities, indicating that it may be a less favourable conformation. C) Rosenthal-Henderson plot, estimating B-factor to be -156.

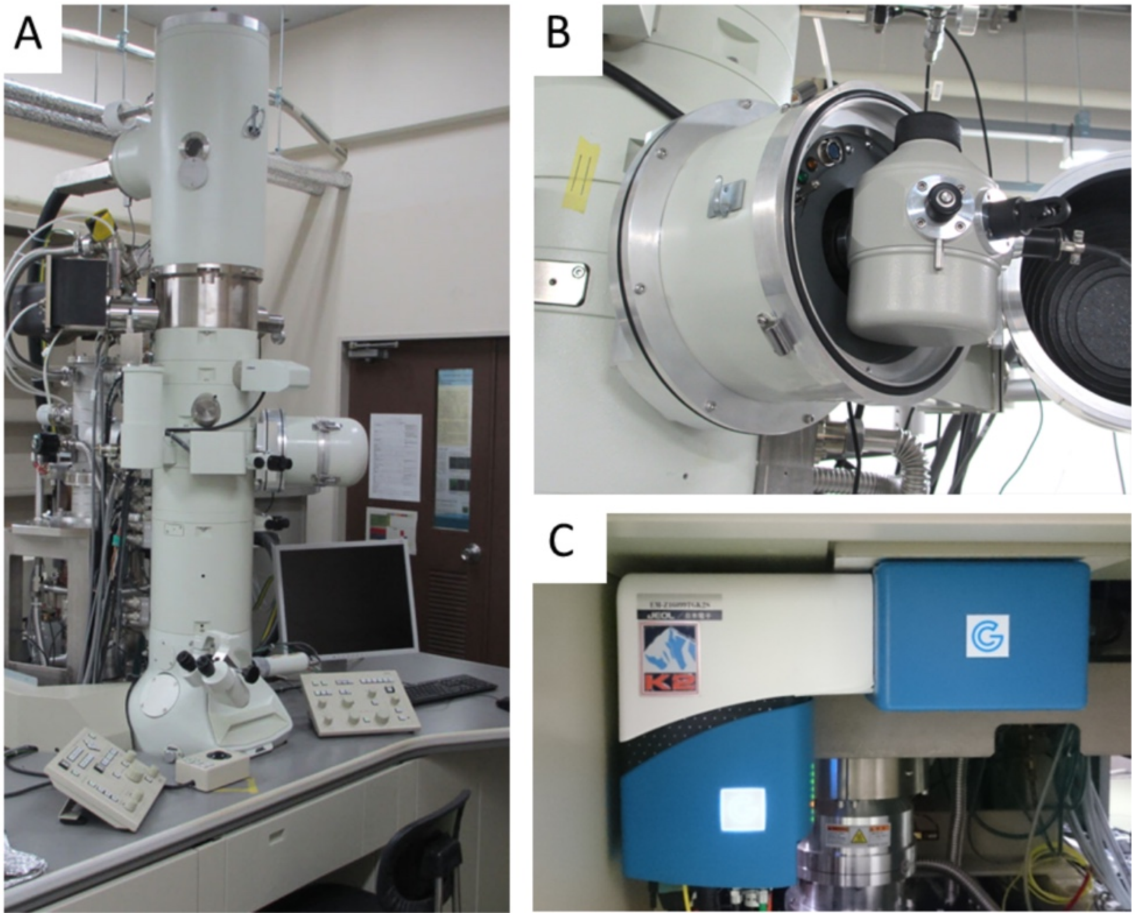

Fig. S2. One of two electron microscope settings (Setting A). Gatan K2 Summit DED (C) is mounted on JEM-2100F microscope (A). Gatan 626 cryo-specimen holder is used to sustain the frozen grid at liquid Nitrogen temperature (B). The second electron microscope setting (Setting B) has been detailed previously (Murata and Wolf, 2018).

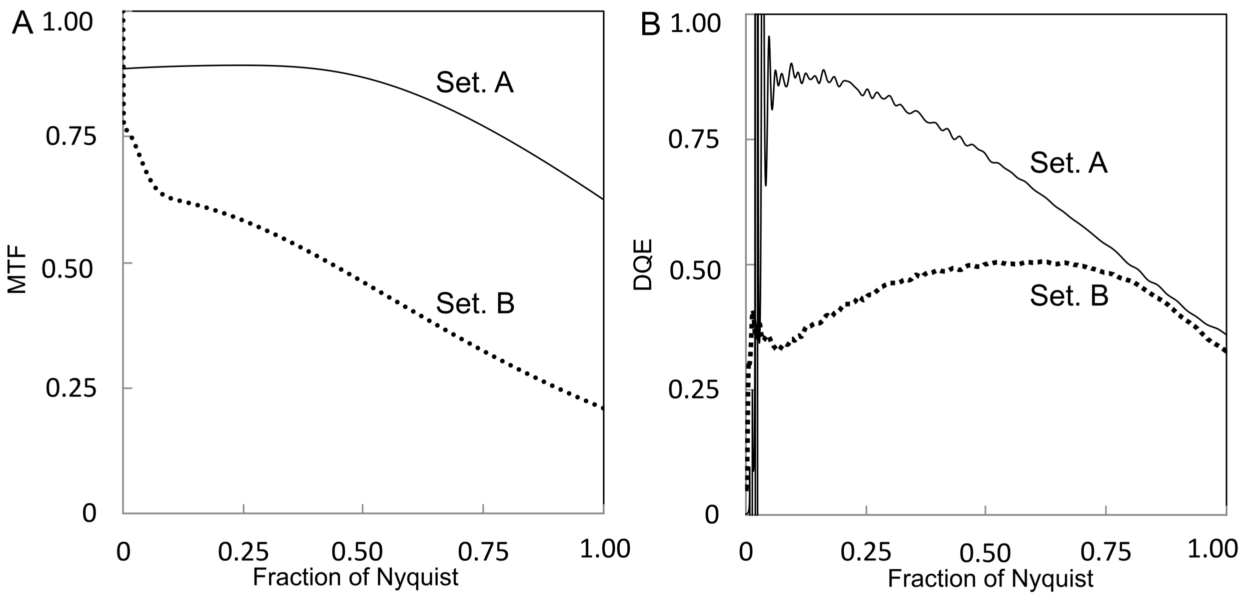

Fig. S3. Modulation transfer function (MTF) and Detective quantum efficiency (DQE) curves in EM settings A and B. A) MTF curves for each setting, B) calculated DQE for each setting. DQE curves are estimated with a beam stopper using FindDQE software (Ruskin et al., 2013).

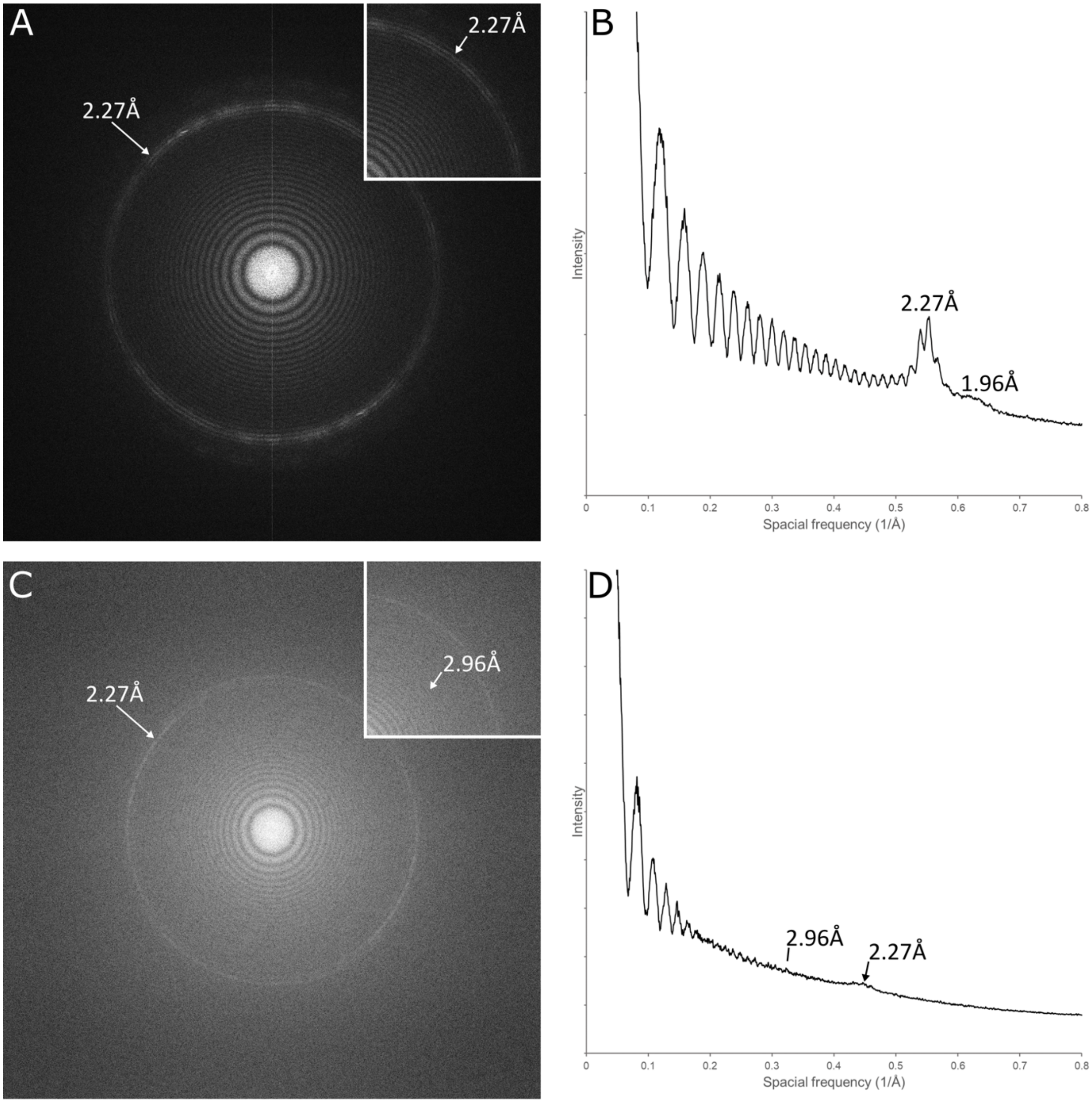

Fig. S4. Thons rings of FFT images of Pt-Ir film. Micrographs of Pt-Ir film were acquired by Setting A and B at 100,000×, 0.5μm defocus, and the power spectra were generated by FFT. A) Setting A power spectrum, B) Plot of rotationally averaged radial profile of (A), C) Setting B power spectrum, D) Plot of rotationally averaged radial profile of (C). With Setting A, Thon rings are clearly distinguishable to the diffraction ring; with Setting B, difficulties in maintaining stability have caused a slight drift in defocus and minor astigmatism resulting in blurring of the rotationally averaged profile.

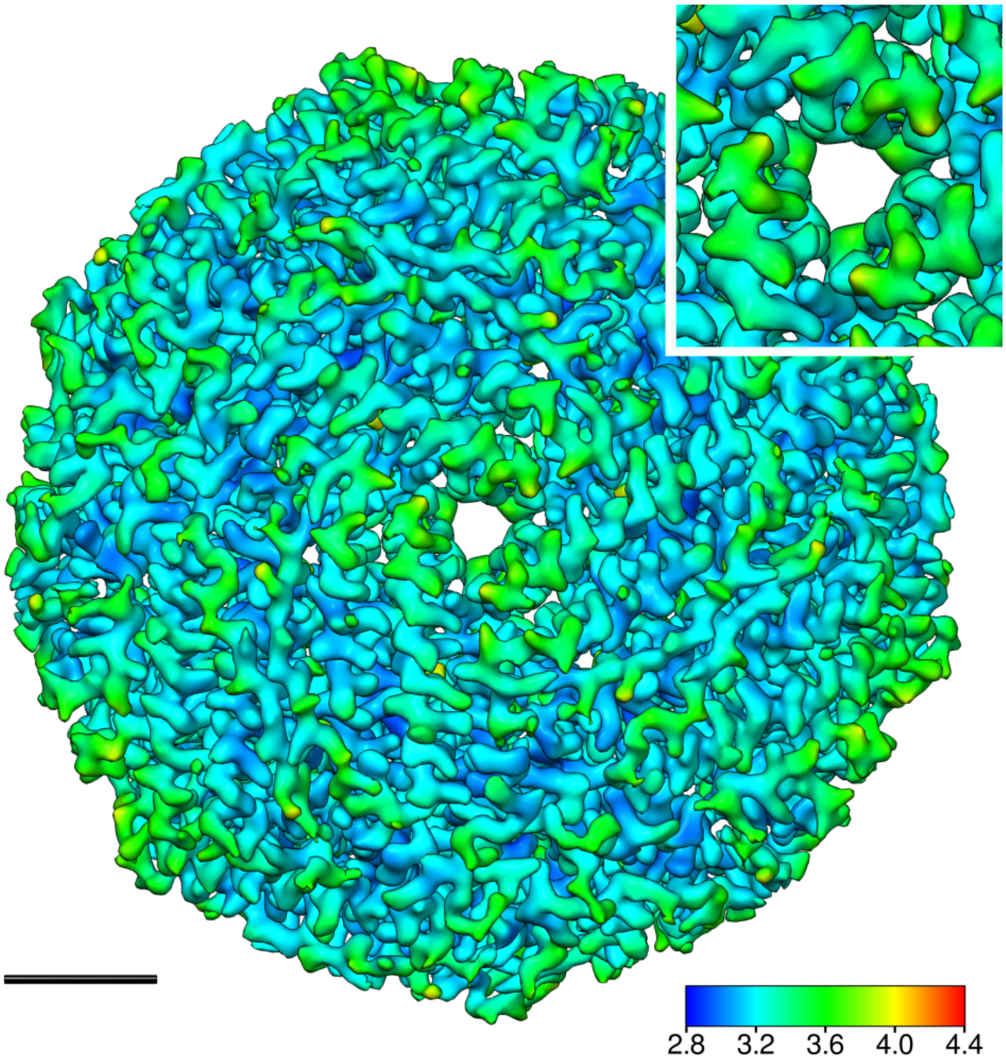

Fig. S5. The best resolution map of apoferritin at 3.3 Å generated by Setting A and using the limited dataset (Table 2). It was achieved at 50,000× magnification. The local resolution was coloured from 2.8 to 3.6 Å resolution. Surface depicted at 4σ. Breakout focussed on 3-fold symmetry axis, where estimated local resolution for each subunit is identical. Scale bar equals 2 nm.

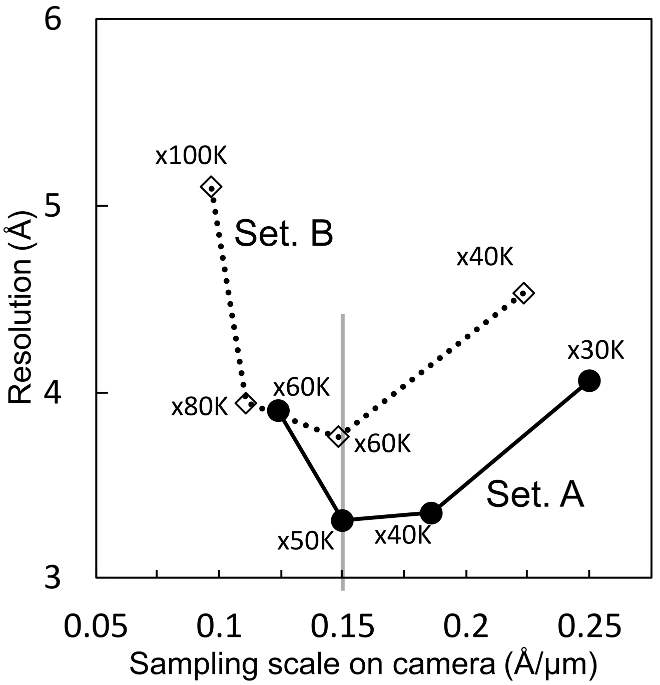

Fig. S6. Resolution plots for sampling scales at detector face. Final global estimated resolution of each reconstruction at different magnifications was generated using Settings A and B. Vertical grey line indicates the scaling point (0.15 Å/µm) at which maximum resolution was achieved for both Settings A and B.
